# Network-centric analysis of co-fractionated protein complex profiles using SECAT

**DOI:** 10.1016/j.xpro.2023.102293

**Published:** 2023-05-12

**Authors:** Benjamin J. Bokor, Darvesh Gorhe, Marko Jovanovic, George Rosenberger

**Affiliations:** 1 Department of Biological Sciences, Columbia University, New York City, NY 10027, USA; 2 Department of Systems Biology, Columbia University, New York City, NY 10032, USA

**Keywords:** Bioinformatics, Proteomics, Mass Spectrometry

## Abstract

The Size-Exclusion Chromatography Analysis Toolkit (SECAT) elucidates protein complex dynamics using co-fractionated bottom-up mass spectrometry (CF-MS) data. Here, we present a protocol for the network-centric analysis and interpretation of CF-MS profiles using SECAT. We describe the technical steps for preprocessing, scoring, semi-supervised machine learning, and quantification, including common pitfalls and their solutions. We further provide guidance for data export, visualization, and the interpretation of SECAT results to discover dysregulated proteins and interactions, supporting new hypotheses and biological insights.

For complete details on the use and execution of this protocol, please refer to Rosenberger et al. (2020).^1^

## Highlights

• SECAT elucidates protein complex dynamics using CF-DIA-MS data
• Tutorial walking through data requirements, parameters, and expected results
• Common pitfalls and potential solutions are explained in detail
• Potential downstream analyses are highlighted and illustrated

## Abstract (editor highlights)

Publisher’s note: Undertaking any experimental protocol requires adherence to local institutional guidelines for laboratory safety and ethics.

## Graphical abstract

**Figure:**
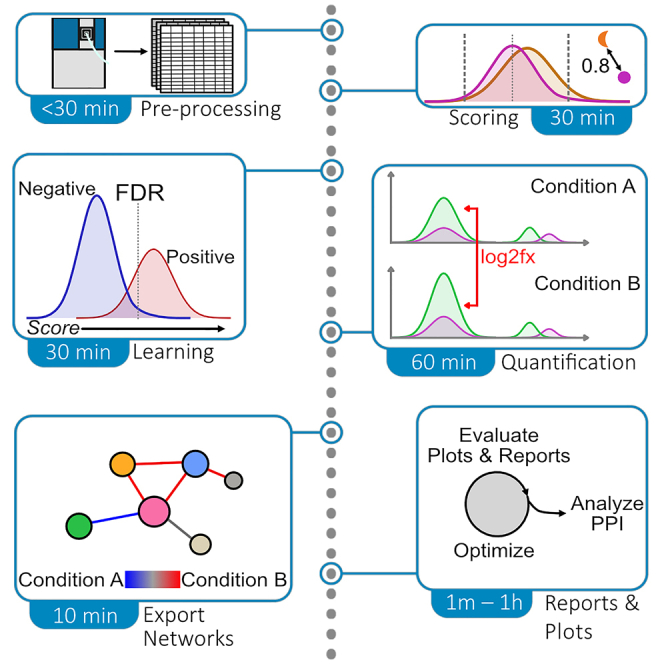

## Before you begin

### Background

The Size-Exclusion Chromatography Analysis Toolkit (SECAT) was designed to identify true protein interactions, quantify differential interactor abundances and to summarize interactions at the protein network level.^1^ The software uses semi-supervised learning on ground-truth true and false or decoy protein interactions to model the confidence of protein interactions in the protein size-separation experiments.^2^ SECAT also provides feedback on the semi-supervised learning performance and interaction identification for gauging data quality. Protein interaction scoring uses a list of target interactions from databases such as STRING^3^ or CORUM^4^ to identify interactions with the proteomic profiles of size-separated fractionated lysates. Alternatively, SECAT can use custom interaction references as an input or discover novel interactions (reference-network-free). As opposed to CCprofiler, which determines complex membership of constituent proteins, SECAT identifies individual protein interactions.^5^ Importantly, SECAT can also quantify the difference in interactions between two conditions. The primary output that SECAT provides are interaction networks with quantitative differences for visualization with tools such as Cytoscape^6^ and quantitative protein-level features that can be used in downstream applications such as gene set enrichment analysis (GSEA).^7^ For specific interactions of interest, fraction-resolved protein abundance plots can be produced to compare interactions between conditions.

***Note:*** Refer to the terms in Table 1 when uncertain about the definition of metrics and data types described in the methodology.

**Table 1:** Common terms and definitions

Term	Definition
target network	protein interaction network whose interactions SECAT will try to detect and quantify
positive network	ground truth positive (expected to be true) prior knowledge or reference protein interaction network (e.g., CORUM or STRING) used to learn SECAT's classifier
negative network/decoys	ground truth negative (expected to be false) prior knowledge or reference protein interaction network (e.g., inverted CORUM or null model) used to learn SECAT's classifier
.secat files	SQLite file storing all preprocessed data and results generated by SECAT
VIPER scores	VIPER normalized enrichment scores (NES) used for the differential statistical tests. VIPER NES represents z-scores, which can be used for other statistical tests, machine learning or visualization. A positive score indicates increased protein abundance (e.g., monomeric, or complex-bound) or interaction dysregulation (e.g., interactor ratio) compared to the reference samples. A negative score indicates decreased effects compared to the reference samples.
log2fx	log2 fold change between case vs. control conditions. A higher number signifies an increase in partial protein abundance or fraction of interaction compared to the control samples.
d-score/score	Discriminant score assigned to each interaction, separating true from false interactions by integration a set of partial scores
interaction pvalue	empirical p-values estimated using the null model interactions
quantitative pvalue adjusted	Integrated p-values (using empirical Brown's method) and multiple testing corrected by the approach proposed by Benjamini-Hochberg (BH)
interaction qvalue	empirical p-values corrected for multiple testing by the approach proposed by Storey-Tibshirani
interaction pep	posterior error probability, estimated by the approach proposed by Storey
lowess normalization	normalization by sliding window lowess smoothing of peptide intensities across the fractions of each experiment/replicate
data-dependent acquisition (DDA)	mode of MS acquisition that targets a set number of the highest MS1 peaks for MS2 analysis
data-independent acquisition (DIA)	mode of MS acquisition that isolates precursors into many windows for convoluted MS2 analysis
pi_0_ lambda	Storey's q-value lambda is a tuning parameter (between 0 and 1) of pi_0_. The higher the lambda value, the more variance is permitted for estimating pi_0_.
pi_0_	Estimated proportion of features (interactions) that are truly null.
protein level: assembled abundance	Statistical metric based on VIPER NES describing changes of assembled protein abundance between case and control samples. This metric sums the protein's intensities above the monomer cutoff and takes the average between both conditions.
protein level: monomer abundance	Statistical metric based on VIPER NES describing changes of monomeric protein abundance between case and control samples. This metric sums the protein's intensities below the monomer cutoff and takes the average between both conditions.
protein level: total abundance	Statistical metric based on VIPER NES describing changes of total protein abundance between case and control samples. This metric sums the protein's intensities throughout the entire gradient and takes the average between both conditions.
PPI level: complex abundance	Statistical metric based on VIPER NES describing changes of complex-bound protein abundance between case and control samples. This metric aggregates both interacting proteins intensities where the two overlap above the monomer cutoff.
PPI level: interactor ratio	Statistical metric based on VIPER NES describing changes of interactor ratio between case and control samples. This metric aggregates the difference between the two conditions of the interacting proteins intensities where they overlap above the monomer region.
PPI level: interactor abundance	Statistical metric based on VIPER NES describing changes of interactor abundance between case and control samples. This metric sums the bait protein's intensities where it overlaps with the prey protein.

### Size separation of lysates

Co-fractionation experimental designs use differential separation of protein complexes (e.g., size and shape in size-exclusion chromatography) to identify differences in complex composition.^8^^,^^9^ Generally, proteins are separated better with increasing number of fractions; however, prior meta-analyses have shown that 40 fractions are frequently sufficient to provide adequate separation between complexes in a SEC run.^9^ SECAT can assess monomeric and assembled fractions separately by inferring a dividing fraction threshold based on each protein’s monomer size. Therefore, SECAT uses a calibrated molecular weight standard to align the size and shape of proteins for co-fractionation analysis. A standard is best run before and after performing separation on lysates, and one should ensure other separation parameters remain constant between conditions/replicates to minimize sources of variation.^5^^,^^10^^,^^11^ For more information regarding potential experimental challenges, please refer to problem 1 in troubleshooting.

1. Format the co-fractionation annotation by creating a ∗.csv file (e.g., in Excel) with the following columns.
  a. run_id: a unique name for each fraction matching the fractions in the mass spectrometry intensity reports (see below) (usually containing information about the sample/condition, replicate, and the fraction number - *ex. Mitosis_replicate2_fraction30* or *mito_2_30*).
  b. sec_id: an integer of the fraction number. The fraction identifiers will be mapped to their corresponding molecular weights for each run_id separately and thus do not need to be aligned between runs.
  c. sec_mw: obtained by extrapolating from a linear fit of the molecular weight standard, each fraction should have an associated molecular weight (refer to the sample sec mw file in sample data’s zenodo repository in the key resources table – location: ‘HEK293-EG/input/hek293_sec_mw.csv’).^11^
  d. condition_id: The name of the condition for a specified sample. SECAT differential analysis utilizes comparisons between two conditions and different samples. This identifier is used later to refer to the samples during analysis.
  e. replicate_id: an integer referring to the replicate number for each condition. The analysis can be run on a single replicate, but this will limit the statistical power of the identified dysregulated interactions.

***Note:*** The protein complex separation can be accomplished through other methods, such as a sucrose column, if there is appropriate separation of the complexes of interest and a monotonic function can attribute a molecular weight to each fraction.

**CRITICAL:** When running a standard and samples on a SEC column, proteins and complexes of a given molecular weight will elute from the column at similar retention times. If problems related to the size of the protein complexes are observed as part of the analysis, there might be an underlying issue with the calibration of MW and fraction number. For example, by not making sure the fraction collector is set to collect at the same retention time for each standard/condition. For more considerations, refer to problem 1 in troubleshooting.

### Mass spectrometry-based protein identification and quantification

The subsequent proteome identification and quantification should be accomplished by a method appropriate for consistent quantitation. For example, SWATH-MS utilizes data-independent acquisition (DIA) to quantify differences between fractions, replicates and conditions.^5^^,^^10^ In our experience, spectral counting, or MS1-based label-free quantities obtained from DDA-MS experiments do not provide consistent data matrices suitable for the peptide-level analysis conducted by SECAT. Therefore, SECAT analysis of DDA-MS data will suffer from lower sensitivity and reproducibility. For more information regarding usage of DDA-MS data, refer to the considerations proposed by Heusel et al.^5^

The MS preparation begins by digesting the fractionated sample and subsequent sample processing, such as desalting, for tandem mass spectrometer (LC-MS/MS).^12^ The mass spectrometry raw data can then be analyzed by quantitative analysis suites, such as OpenSWATH,^13^ Skyline,^14^ DIA-NN,^15^ Spectronaut,^16^ EncyclopeDIA^17^ or other tools. Note that some analysis software perform operations such as matching between runs, background inferences and re-quantification – in these cases SECAT users should consider *detrending* in the scoring step (Figure 2).

### Retrieving and formatting input data

2. SECAT requires a positive and a negative reference protein interaction network to train a PPI classifier, a target network for identifying interactions of interest within the experimental conditions, a UniProt metadata XML file to annotate the identified proteins, and a quantitative, peptide-level matrix with each fraction per condition/replicate. The quantitative matrix can be provided as a TSV/CSV file in a matrix format (rows: protein IDs and corresponding peptides, columns: runs, values: peptide intensities) or long list (columns: peptide_id, protein_id, run_id, peptide_intensity) formats with the following columns:
  a. peptide_id: the peptide sequence or precursor identifier reported by the primary analysis software.
  b. protein_id: the majority UniProtKB protein identifier that is associated by the primary MS analysis software. Only proteins based on unique, non-shared, proteotypic peptides are currently supported. The identifiers need to correspond to the UniProtKB metadata XML file. Note that each peptide should be associated with a single UniProtKB identifier – SECAT does not support protein group IDs. If selecting a single UniProtKB identifier becomes a source of uncertainty, refer to problem 2 in troubleshooting.
  c. run_id: The MS run identifier mapping to the experimental design file as described above.
  d. peptide_intensity: The reported peptide-level intensity from sequencing analysis software such as OpenSWATH, MaxQuant, Spectromine/Spectronaut.
3. Download UniProtKB metadata XML file for protein annotation.
  a. Refer to Uniprot.org.
  b. Search for the organism of interest (the parsing algorithm has been confirmed with human, mouse, and yeast).
  c. Download as a compressed XML file (.xml.gz).
4. Download protein interaction/complex database file for protein interactions identification.
  a. SECAT takes multiple networks as inputs (see Table 2 for considerations):
    i. Target interaction network is optional but strongly recommended (e.g., STRING). Without a target network all combinatorial protein interactions will be assessed and have a negative effect on sensitivity.^1^ For untargeted/novel interaction no network should be input.
    ii. Positive interaction network is also optional but strongly recommended. Ideally a well-established network such as CORUM should be used to model true positive interactions in humans. Non-human organisms will require finding confident interactions specific to that organism in other databases (see key resources table). In less common organisms, one should have at least around 10,000 confirmed interactions in the positive interactions network for the learning step to be effective. If not specified, then SECAT will try to use the top scoring PPIs of the target network as a positive network.
    iii. Negative interaction network is also optional but strongly recommended. A limited set of off-target non-CORUM interactions are provided in the Zenodo repository for human interactions (refer to Table 2). For other model systems, a negative network could be generated following these steps: First, a high-confidence protein complex interaction network, where complex structure is available for most subunits, should be selected as reference (e.g., CORUM or AlphaFold-Multimer). Importantly, the subunits are expected to not interact with other subunits in the dataset. To obtain the negative network, the inverse set of interactions of these subunits is computed. To further remove false positives, known and predicted transient interactions from other databases or predictions (e.g., STRING) should be excluded. If a negative network can’t be obtained or generated, a randomly permutated decoy network can be generated by SECAT, although we found that this resulted in lowered sensitivity. Generally, we have found that the negative network should be around twice as large as the positive network.

**Table 2:** Network input considerations

	Sensitivity & selectivity	Identification of novel interactions	Detectability of differential complex dynamics	testNet (PPIs of interest are known)	posNet (solid reference of known complex-like PPIs, e.g., CORUM)	negNet (solid reference of known unlikely PPIs, e.g., inverted CORUM)	Reported number of covered proteins	Reported PPI number estimate (q < 0.05)	Reported number of net-dysregulated proteins (q < 0.05)
Targeted analysis of well characterized model systems	+++	no	+++	yes	yes	yes	5091	8573	529
Untargeted analysis of well characterized model systems	+	yes	++	no	yes	yes	5091	2212	313
Targeted analysis of poorly characterized model systems	++	No	++	yes	no	no	5091	5872	498
  b. Refer to the key resources table for established networks. Human, mouse, and yeast systems have currently been tested. The Zenodo repository contains target, positive, and negative networks that have been used on the sample data in this paper.
  c. For targeted studies, a two-column data frame of interactions can be input as the network.

**CRITICAL:** An easy mistake users can make is mislabeling columns and fraction names. The names of the fractions in the peptide intensity file must match those in the co-fractionation annotation file. Either a single table or multiple tables can be used for the peptide-level intensities, so long as the fraction names are uniquely matching the co-fractionation annotation file. We suggest first time users to refer to the sample data (key resources table). Alternatively, the --column parameter can be used during data import to specify the name of the columns (refer to data preprocessing). Failure to correctly assign columns will result in the issue presented in problem 3 in troubleshooting.

### Installing SECAT

Install the SECAT software before running the performing the analyses described in the protocol.

***Optional:*** For first time installations, it recommended to use anaconda and to work in a virtual environment. For users familiar with Docker, SECAT can be downloaded as a preconfigured Docker container. Due to the high-memory usage of SECAT, Docker users should ensure that the container is run with at least 16GB of memory. You can find instructions for downloading Docker for your machine here. On Mac OS and Windows, you will need Docker desktop. On Linux, running the Docker daemon should be sufficient.

5. Installation.
  a. Conda.
    i. Create a conda environment with only Python, numpy, and pip and call it secat.
      > conda create -n secat python==3.10.8 numpy pip -y
    ii. Activate the secat conda environment.
      > conda activate secat
    iii. Install the secat python package via pip and its dependencies.
      > pip install secat
    iv. Check installation by running help.
      > secat --help
  b. Pip (no conda).
    i. Install via pip into your default python environment.
      > pip install secat
  c. Docker.
    i. Pull the Docker container.
      > docker pull grosenberger/secat:latest
    ii. Check the container is working properly.
      > docker run --name secat --rm -i -t grosenberger/secat:latest secat -–help
    iii. To run analyses, create a folder to mount onto the container. This folder should contain your input files and a shell script which will trigger the SECAT commands (see step 2 for an example).
6. Retrieving Example Data
  a. Download the HeLa-CC.tgz and Common.tgz files from Zenodo Repository: https://doi.org/10.5281/zenodo.3515927 into your SECAT working directory.
  b. Unzip the tgz files.
    i. Recommended way:
      > tar -xzvf HeLa-CC.tgz
      > tar -xzvf Common.tgz
    ii. Alternatively, use any compression utility that supports compressing and decompressing gzip and tarball files.
7. Run SECAT on Example Data.
  a. Conda:
    i. Activate conda environment.
      > conda activate secat
    ii. Navigate inside the HeLa-CC/ folder from step 2.
    iii. Launch the secat.sh script via:
      > bash secat.sh
  b. Local secat installation (non-conda):
    i. Navigate inside the HeLa-CC/ folder from step 2.
    ii. Launch the secat.sh script via:
      > bash secat.sh
  c. Docker:
    i. Combine the HeLa-CC and Common folders into one folder called data.
    ii. Navigate to the parent folder of data/.
    iii. Run SECAT in the Docker container by mounting the data folder.
      > docker run --name secat --rm -v $PWD/data:/data -i -t grosenberger/secat:latest cd /data/HeLa-CC && bash secat.sh

## Key resources table

**Table:** 

REAGENT or RESOURCE	SOURCE	IDENTIFIER
**Software and algorithms**		

Anaconda	Anaconda, Inc.	https://docs.anaconda.com/anaconda/install/index.html
Cytoscape	Shannon et al.^6^	https://cytoscape.org/
SECAT	This paper^1^	https://github.com/grosenberger/secat https://doi.org/10.5281/zenodo.7799334
PyProphet	Teleman et al.^18^	https://github.com/PyProphet/pyprophet
decoupler (VIPER)	Alvarez at al.^19^ & Badia-i-Mompel et al.^20^	https://github.com/saezlab/decoupler-py
CCprofiler	Heusel et al.^5^	https://github.com/CCprofiler/CCprofiler
aLFQ	Rosenberger et al.^21^	https://cran.r-project.org/web/packages/aLFQ/index.html
AutoAnnotate	Kucera et al.^22^	http://apps.cytoscape.org/apps/autoannotate

**Deposited data**		

Sample Data (Zenodo)	This paper^1^	https://doi.org/10.5281/zenodo.3515927
Cytoscape Walkthrough	This paper	https://doi.org/10.17632/xkrrw8xtnj.1
PrePPI	Zhang et al.^23^^,^^24^	http://honig.c2b2.columbia.edu/preppi
CORUM	Giurgiu et al.^4^	http://mips.helmholtz-muenchen.de/corum/
BioGRID	Stark et al.^25^	https://downloads.thebiogrid.org/BioGRID/Release-Archive/BIOGRID-4.4.204/
BioPlex	Huttlin et al.^26^^,^^27^^,^^28^	https://bioplex.hms.harvard.edu/interactions.php
STRING	Szklarczyk et al.^3^	https://string-db.org/cgi/download?sessionId=btyrFftVuyW0
UniProt	UniProt Consortium^29^	https://www.uniprot.org/proteomes/
IID	Kotlyar et al.^30^	http://iid.ophid.utoronto.ca/
IntAct	Orchard et al.^31^	https://www.ebi.ac.uk/intact/home
Hu.MAP 2.0	Drew at al.^32^	http://humap2.proteincomplexes.org/
HuRI	Luck et al.^33^	http://www.interactome-atlas.org/

## Materials and equipment

• Quantitative peptide-level proteomic data with associated majority protein ID.
• Co-fractionation experiment MW calibrated to each fraction.
• UniProt metadata XML file.
• Interaction Databases: target network, positive network, and negative network (as many as possible).

## Step-by-step method details

The following steps will show blocks of code for use through terminal (for windows one can install anaconda and use cmd.exe, windows terminal app, or git-bash for linux). The data and files used below are retrieved from the Zenodo link above. The estimated times given assume 16GB RAM, Intel i7 8-cores, and the sample data. Note that the software requires around 16GB of RAM.

### Data preprocessing

**Timing: <30 min**

**Figure 1:**
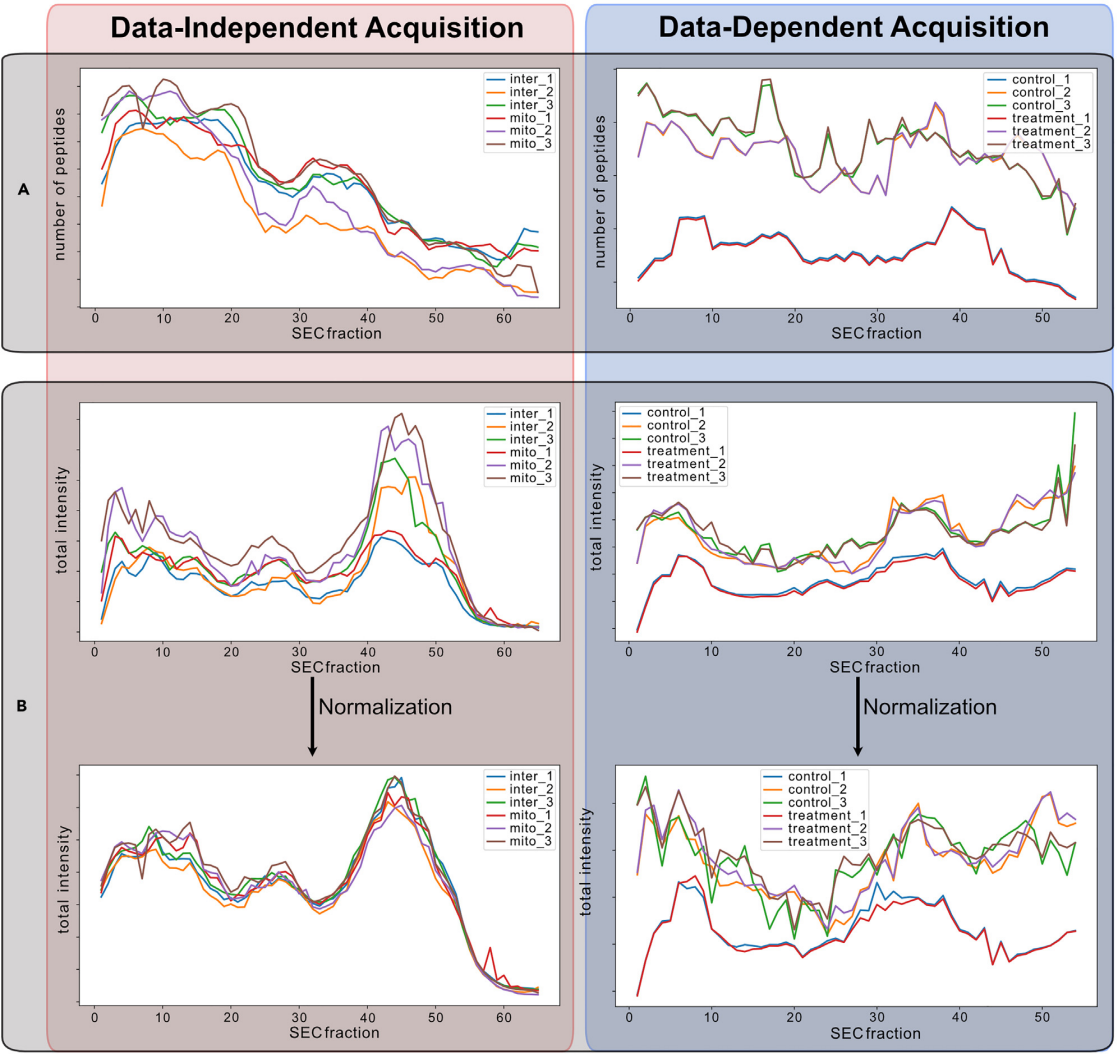
Lowess normalization and coverage SECAT performs a lowess normalization during preprocessing. (A) Plots of the peptide counts for two SECAT runs. A set of DIA experiments on the left (red) and a set DDA experiments on the right (blue). The normalization relies on the peptide counts, and the DIA experiment counts are relatively consistent while the DDA experiment counts differ drastically between experiments. (B) The normalization of the DIA data (left, red) successfully smooths the data between the experiments. However, the DDA experiment normalization has a negative effect on the smoothness of the data. DDA experiments often differ widely in peptide counts between experiments and fractions; therefore, SECAT users should consider whether their experiment should use normalization by viewing the normalization plots.

The first SECAT command reads the input files and propagates the information to SECAT’s SQLite database. If the normalize option is true, this step will perform a cyclic LOWESS normalization of the peptide intensities over the replicates and conditions, via a sliding window of the fractions. This step can account and normalize for some variability, for example arising from heterogeneous sample amounts measured by label-free quantification, but not for differences in proteome coverage or SEC gradients between replicates and conditions. A successful normalization results in overlapping total protein abundance profiles over the SEC fractions for all conditions and replicates, changing the total amplitude, but not the underlying distribution (Figure 1A). In contrast, unaligned runs due to different proteome coverage or an unaligned SEC fractionation process will result in altered distributions of conditions and replicates (Figure 1B).

1. Prepare and move the files listed in the materials and equipment section into a folder.
  a. Minimum requirements: (1) peptide intensity file, (2) sec (co-fractionation) mw file, (3) uniprot annotation file.
  b. First time users are suggested to use the sample data and run the blocks of example code.

2. Run the data preprocessing step by inputting the parameters in terminal-related interface.
  a. Example code from sample data:
    >secat preprocess
    --out=hela_string.secat
    --sec=input/hela_sec_mw.csv
    --net=common/9606.protein.lnks.v11.0.txt.gz
    --posnet=common/corum_targets.txt.gz
    --negnet=common/corum_decoys.txt.gz
    --uniprot=common/uniprot_9606_20190402.xml.gz
    --min_interaction_confidence=0
    input/pep∗.tsv
  b. Required/Important Parameters:
    --out PATH
    ***Note:*** [Required] The SECAT file is an SQLite database that is saved as ‘.secat’ file.
    SECAT with a previous ‘.secat’ file will override the current data from the previous run while still maintaining the data structure.
    --sec PATH
    ***Note:*** [Required] A table input of the SEC calibration. Refer to the example data for its structure. The five columns are: run_id, sec_id, sec_mw, condition_id, and replicate_id. The run_id must match the names of the intensity file columns. This file may be edited to fit other co-fractionation methods besides SEC if a MW can be ascribed to each fraction.
    --net PATH
    ***Note:*** [Optional but recommended] A reference network file for querying interaction targets in the protein profiles. Currently, SECAT supports querying networks in miTAB, STRING DB, BioPlex, and PrePPI formats. The network formats are identified by their column names. Also, a custom target interaction network can be input as well. The target network should be formatted with two columns: one for each protein participating in the interactions. When there is no attached posnet (true positive interaction network) or negnet (true negative interaction network) the net file is used to create a positive and negative network for the semi-supervised learning. Once SECAT has trained a model, the net file is then used for identifying PPI interactions in the samples. Only interactions listed in this file will be identified and quantified for later analysis. If this parameter is not included, all possible protein interactions will be targeted.
    --posnet PATH
    ***Note:*** A positive binary interaction reference file. The interactions will be used as the true positives in the semi-supervised learning by PyProphet when generating the classifier. If a positive network is not included, one will be generated from the target network. The data should be formatted as described above for the ‘net’ input. For an example, refer to the positive CORUM network in the Zenodo repository.
    --negnet PATH
    ***Note:*** A negative binary interaction reference file. The interactions will be used as the false interactions in the semi-supervised learning by PyProphet when generating the classifier. If a negative network is not included, one will be generated from the positive network. As previously, refer to the above ‘net’ input format and/or the negative CORUM network from the resources.
    --uniprot PATH
    ***Note:*** [Required] UniProt metadata XML file for molecular weight reference (can also be formatted as an xml.gz file). For the best results use the latest UniProt file including the extended TrEMBL entries. In general, the corresponding XML file to the primary mass spectrometric analysis FASTA database should be used.
    ***Note:*** UniProt XML parsing works directly on Human, Mouse, and Yeast. However, other organisms may have slightly different UniProt formats that may require parsing modifications. Refer to problem 4 in troubleshooting for details.
  c. Other parameters.
    --columns COLUMN_1 COLUMN_2 … COLUMN_9
    ***Note:*** The 9 necessary columns and their order is as follows: [default: run_id, sec_id, sec_mw, condition_id, replicate_id, run_id, protein_id, peptide_id, peptide_intensity]. Use this feature to specify alternative primary analysis software report header names. E.g., for Spectronaut reports, this parameter could be set to --columns run_id sec_id sec_mw condition_id replicate_id R.FileName PG.ProteinAccessions EG.PrecursorId FG.Quantity. Input the name of your columns in the order that they match to the default show above. The order of the column names matters.
    --normalize / --no-normalize
    ***Note:*** Normalize quantification data by sliding window cycling LOWESS normalization between replicates, conditions, and SEC fractions [default: normalize].
    --normalize_window INTEGER
    ***Note:*** Number of SEC fractions per sliding window [default: 5]. This parameter should be changed if the number of fractions and resolution of the fractionation change dramatically from the example SEC setup. In the sample data, elution peaks span around 10 fractions. Increase the normalization window if your peaks will span significantly more than this.
    --normalize_padded / --no-normalize_padded
    ***Note:*** Use padding for first and last SEC fractions to ensure that each fraction is covered by an equal number of windows [default: normalize_padded]. Only consider disabling this option if the sliding window is almost as large as the total number of SEC fractions.
    --decoy_intensity_bins INTEGER
    ***Note:*** Number of decoy bins for intensity, only used if no negative network is supplied [default: 1]. The number of decoy bins can be tweaked to increase the likelihood of proteins with similar elution profiles to be randomly selected as null data point. This option could be tweaked for experimental designs substantially differing from the SEC experimental design, but generally does not need to be configured.
    --decoy_left_sec_bins INTEGER
    ***Note:*** Number of decoy bins for left fraction of an SEC elution peak (start of peak), only used if no negative network is supplied [default: 1]. The number of left decoy bins can be tweaked to increase the likelihood of proteins with similar elution profiles to be randomly selected as null data point. This option could be tweaked for experimental designs substantially differing from the SEC experimental design, but generally does not need to be configured.
    --decoy_right_sec_bins INTEGER
    ***Note:*** Number of decoy bins for right fraction of an SEC elution peak (end of peak), only used if no negative network is supplied [default: 1]. The number of right decoy bins can be tweaked to increase the likelihood of proteins with similar elution profiles to be randomly selected as null data point. This option could be tweaked for experimental designs substantially differing from the SEC experimental design, but generally does not need to be configured.
    --decoy_oversample INTEGER
    ***Note:*** Number of iterations to sample decoys from the interaction targets if ‘negnet’ is not included [default: 2]. The decoy query generation function shuffles the bait_id from the net_data input (or the posnet_data when present). The number of decoys will increase as the decoy oversample parameter increases. We have found that a larger number of decoys improves the accuracy of the classifier.
    --decoy_subsample / --no-decoy_subsample
    ***Note:*** Decoys generation is subsampled to approximately the same number of targets queries [default: no-decoy_subsample]. Use this parameter with care. Generally, more decoys are beneficial to the classifier performance. However, this can be useful if there is an overwhelming number of decoys that are negatively affecting the error-rate estimation step.
    --min_interaction_confidence FLOAT
    ***Note:*** Minimum interaction confidence for reference interaction databases. Use this parameter to filter out interactions in networks that include a confidence score for each entry [default: 0.0] (E.g., probability > 0.9 for STRING, and probability > 0.5 for PrePPI).
    --interaction_confidence_bins INTEGER
    ***Note:*** Number of interaction confidence bins for grouped error rate estimation if a reference interaction confidence value is provided for the target network [default: 100]. When used with reference interaction networks like STRING DB or PrePPI, which provide a confidence score for different categories of interactions, higher likelihood PPIs will be scored in separate groups than lower likelihood PPIs, thus increasing the sensitivity.
    --interaction_confidence_quantile / --no-interaction_confidence_quantile
    ***Note:*** Whether interaction confidence bins should be grouped by quantiles [default: interaction_confidence_quantile]. If the prior confidence scores should be considered as a confidence ranking score, rather than as an accurate distribution of the score, grouping interactions by quantiles can be more robust.
    --use_cached_uniprot True/False
    ***Note:*** Stores a parquet file of the parsed Uniprot file on disk to reduce preprocessing time for future runs. The file is stored with the same base name as the input Uniprot file from part f. and in the same directory. This parameter is intended to reduce the memory footprint of the data preprocessing step and to reduce execution time overall. The same Uniprot file will yield the same table internally so there is no benefit to re-parsing the file during each run. Caching is not the default so users must explicitly include the flag to utilize Uniprot file caching.
    --help
    ***Note:*** Shows brief description of parameters.

3. Check the normalization of the data and continue to signal processing.

***Note:*** Low/Empty intensity fractions often overcorrect and disrupt normalization. Overcorrection is easily detected by viewing the normalized plot PDF reports and can be overcome by removing or correcting empty fractions. In our experience, removing empty/low intensity fractions in only the problem condition/replicate will usually resolve this issue. Refer to problem 5 in troubleshooting for details.

### Signal processing (scoring)

**Timing: ∼30 min**

**Figure 2:**
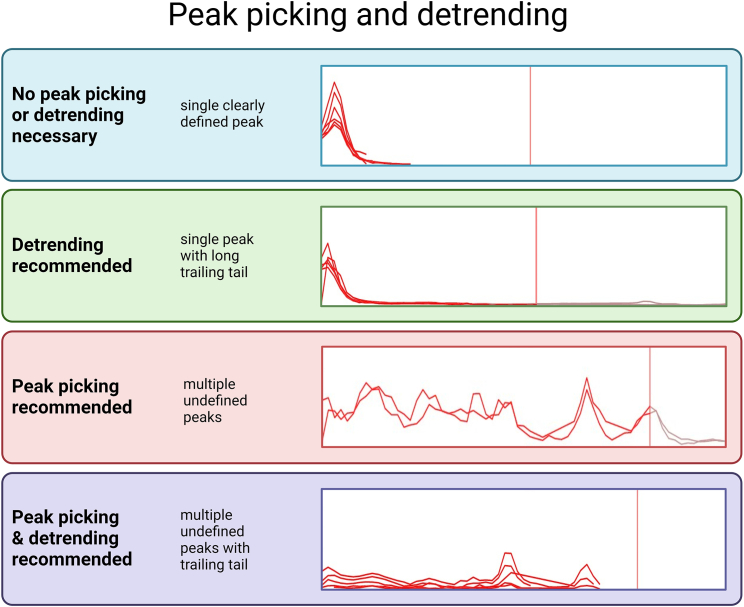
Peak picking and detrending SECAT provides the options to choose peak picking or detrending of the peptide intensities when scoring interactions. Depending on how the users data appears and their interactions of interest, different peak picking and detrending options are recommended. Each panel contains the recommended peak picking and detrending parameter, a description of the peaks, and an example of the description from the sample data.

This step scores the interactions over the complex mass range according to interactions from the protein interaction reference networks. Each protein’s peptide elution profiles are used to compute a score for signal cross-correlation, elution coverage, and other criteria that can be used by the classifier in the next step to differentiate true from false interactions. By default, the scoring in SECAT does not use any peak picking methods, but certain data types may benefit from the following parameters (Figure 2).

***Note:*** Depending on the type of input data, different peak picking and detrending options may improve analysis. The recommendations provided are geared toward maximizing the number of PPI in the sample SEC-SWATH-MS data. SWATH-MS and DIA data analyzed with programs such as OpenSWATH (without “re-quantification”, such as optionally conducted by tools such as TRIC or DIAlignR) generally produce single clearly defined peaks and do not require any sort of peak picking or detrending. DIA data analyzed with “re-quantification” or match-between-runs (MBR) by programs such as Spectronaut or DIA-NN that provide quantitative background DIA signals will often produce peaks with long tails of low intensity. Such data can typically benefit from detrending. In contrast, data with multiple undefined peaks will likely benefit from peak picking and/or detrending.

However, these recommendations may not hold true for all protein interactions or co-fractionation methods. For users interested in specific protein interactions, it is best to look at the elution profiles of the proteins of interest to determine which type of profile is produced when determining the peak picking method. Furthermore, other (untested) separation methods than SEC may provide different elution patterns, and we recommend such users to test different peak picking and detrending options to determine which is a best fit for their data. A simple benchmark that users can utilize in their own optimization is to look at overall numbers of recovered interactions or simply check whether their protein of interest is recovered.

4. Run the scoring step using the output SECAT file from the data preprocessing step and select the necessary parameters.
  a. Example code from sample data:
    >secat score --in=hela_string.secat --threads=8
  b. Required/Important Parameters:
    --in PATH
    ***Note:*** [Required] Use the data preprocessing out SECAT file as the input for this step.
    --peakpicking [none | detrend_zero | detrend_drop | localmax_conditions | localmax_replicates]
    ***Note:*** The method for peak picking of the peptide chromatograms: "none", "detrend_zero", “detrend_drop", "localmax_conditions" or "localmax_replicates" [default: none]. None: profiles with distinct high confidence peaks do not require peak picking (e.g., OpenSWATH without “requantification”).^34^Detrend_zero: averages over all fractions and will set missing peptide identities to zero. If there is a baseline noise level, then a detrend option should be used (e.g., from imputed quantities, Spectronaut / OpenSWATH re-quantification, matching-between-runs, or background inferences). Detrend_drop: averages over all fractions with identified peptides only. If there is a baseline noise level, then a detrend option should be used (e.g., from imputed quantities, Spectronaut / OpenSWATH re-quantification, matching-between-runs, or background inferences). Localmax_conditions: averages peak picking over replicates of the same conditions. Localmax_replicates: conducts peak picking for all samples separately. Consider using if analysis software uses matching between runs, background inferences and re-quantification.
  c. Other Parameters.
    --out PATH
    ***Note:*** Output SECAT file. Use the out PATH to save this part of the analysis in a new SQLite database. Otherwise, the default is to save the output in the --in PATH.
    --monomer_threshold_factor FLOAT
    ***Note:*** Factor threshold to consider a feature a complex rather than a monomer [default: 2.0]. The default threshold of 2.0 excludes any interactions smaller than a homodimer, which are extremely frequent. Decreasing this number will include interactions smaller than the homodimer. Future implementations could include structural predictions of proteome-wide homodimers.
    --minimum_peptides INTEGER
    ***Note:*** Minimum number of peptides required to score an interaction [default: 1]. The default is set to 1 because we have found that including proteins with only one identified peptide does not negatively affect SECAT’s performance. The proteins are quantified by using the peptides with the highest overall intensity.
    --maximum_peptides INTEGER
    ***Note:*** Maximum number of peptides used to score an interaction [default: 3]. Selects only the top N most intense peptides per protein. The lowest intensity peptides are often outside of the linear dynamic response of intensity vs. abundance, and their inclusion will result in non-specific association of potential interactors.
    --chunck_size INTEGER
    ***Note:*** Chunk size for processing [default: 50000]. Ensures that large analyses don’t run out of memory. SECAT was originally configured with 16GB RAM and this parameter can be increased if more memory is available. Computers with lower RAM will benefit from smaller chunks but will be slower due to more read/write operations. However, computers with higher RAM capacity will benefit from larger chunk sizes.
    --threads INTEGER
    ***Note:*** Number of threads/CPU to use for parallel processing. -1 means all available CPUs [default: -1].
    --help
    ***Note:*** Shows brief description of parameters.

5. Proceed to the PPI detection step for SECAT to assess the scores provided in this step.

### PPI detection (learning)

**Timing: ∼30 min**

This step uses scores from the previous signal processing step to generate a confident interaction network. The positive and negative networks from reference databases (or optionally derived from the target network) are used to train a classifier in a semi-supervised fashion over multiple iterations by a gradient-boosting approach. This classifier is then used to assign a confidence score to the interactions in the query space, and then to estimate confidence scores for each interaction. While it is recommended to use a target network along with positive and negative networks for creating a classifier, these parameters are not required. The impact of using all or some of these parameters is shown below (Table 2). Analyses of poorly characterized systems often do not have reference protein-interaction networks associated. In these cases, SECAT analysis will be limited in modeling true interactions and the number of detected interactions will be reduced accordingly.

***Note:*** The initial input of networks in the preprocessing steps will have varying results for the scoring and learning steps of the analysis. In general, adding a net file will produce better recovery due to the higher confidence in interactions, but will limit the discovery of novel interactions. Using a well characterized model with posnet and negnet files will improve the classifiers’ ability to distinguish between true interactions and false positives, but this data is limited to well-characterized model organisms.

6. Run the learning step using the output SECAT file from the scoring step and select the necessary parameters.
  a. Example code from sample data:
    >secat learn --in=hela_string.secat --threads=5
  b. Required/Important Parameters:
    --in PATH
    ***Note:*** [Required] Input SECAT file from previous step.
    --pi0_lambda <FLOAT FLOAT FLOAT>
    ***Note:*** To compute confidence scores for each interaction, PyProphet needs to estimate the proportion of truly absent interactions (pi0) that still result in a partially overlapping signal using the lambda tuning function, proposed by Storey & Tibshirani.^35^ The default start, end and step values for the lambda function are defined as three parameters separated by an empty space. [default: 0.01, 0.5, 0.01] Alternatively, a fixed value can be set as described below:
    i. <START END STEPS>, e.g., 0.1, 1.0, 0.1 (set as: 0.1 1.0 0.1).
    ii. set to fixed value, e.g., 0.4 (set as: 0.4 0 0).
      ***Note:*** For high proteome coverage, high quantitative consistency datasets as the provided examples, this parameter typically does not need to be modified. For some scenarios, it might be useful to enforce a more conservative estimate of pi0 by setting a fixed value (e.g., 0.4) or by limiting the range of lambda (e.g., from 0.01–0.1). For debugging purposes, but not production-level applications (e.g., to visualize the data using SECAT) the lambda function could also be set to an extremely low value (e.g., 0.01 or 1e-6), which result in a pi0 estimate close to 1.0, thus assuming that almost all candidate PPIs result in false signals. A detailed description of the assumptions and considerations can be found in Fig. 3 of Storey & Tibshirani (2003).^35^ For more details refer to problem 6 in troubleshooting.
      ***Note:*** Lower pi0_lambda values are more lenient. Reports are also generated for the learning process that can be used to assess success.
      ***Note:*** If semi-supervised learning in PyProphet fails at this step, failed tuning of the lambda function is typically not the issue but rather the symptom. Most often, the quantitative consistency or coverage of the dataset is not sufficient for SECAT. In this case, manual inspection and data quality assessment as described previously is recommended.^12^
      --test / --no-test
      ***Note:*** Run in test mode with fixed seed to ensure reproducibility [default: no-test]. Set as ‘test’ when comparing parameters to maintain reproducibility.
  c. Other Parameters.
    --out PATH
    ***Note:*** Output SECAT file. Use the out PATH to save this part of the analysis in a new SQLite database. Otherwise, the default is to save the output in the --in PATH.
    --apply_model PATH
    ***Note:*** Apply pretrained SECAT model by supplying the output SECAT file from a previous “secat learn” run. This mode is primarily useful if a classifier should be trained on a different dataset than the one investigated. However, it is important that the general properties between the datasets are conserved (e.g., fractionation resolution, proteome coverage, etc.). This function could be useful if learning is not possible on one dataset (e.g., if no reference interaction networks are available for organism X, but a different dataset with similar properties exists for organism Y). If used, all statistical ‘secat learn’ parameters should be set identically to the modeled data.
    --minimum_abundance_ratio FLOAT
    ***Note:*** Minimum abundance ratio required to score an interaction [default: 0.1]. The default of 0.1 excludes protein interactions with a bait/prey abundance ratio >1:10. This excludes many likely false interactions since protein complexes typically have more similar subunit ratios. This parameter should be changed in analyses where this is not expected to be the case.
    --maximum_sec_shift FLOAT
    ***Note:*** Maximum lag in SEC units between interactors and subunits for an interaction to be identified [default: 10.0]. This parameter should only be changed if the analysis has peaks with a resolution much smaller or larger than the sample data. Ensures that the var_xcorr_shift is less than or equal to this number. The var_xcorr_shift is calculated for each bait/prey pair by finding the apex of correlation between the two and finding the greatest difference between the bait or prey mean. For more information refer to the mProphet paper.^2^
    --cb_decoys / --no-cb_decoys
    ***Note:*** Use decoys only from same confidence bin instead of full set for training the classifier [default: no-cb_decoys]. This option can be used in combination with the decoy binning option in the preprocess step. In that case, selecting this parameter will increase the comparability of decoys to targets to generate a more confident classifier. However, the decoy binning approach should not be used be used if other options are available.
    --xeval_fraction FLOAT
    ***Note:*** Data fraction used for cross-validation of semi-supervised learning step. This parameter feeds into PyProphet [default: 0.8]. This parameter can scale with the size of the input data. Cross-validation needs to subset different portions of the data, and the larger the data set, the smaller the required fraction of data. SECAT was optimized on 3 replicates for 2 conditions but increasing the number of replicates and conditions will allow one to reduce the cross-validation fraction to speed up the learning step and may generate a more robust classifier. To learn more about the cross-validation and the ‘xeval’ functions, refer to previous work.^2^
    --xeval_num_iter INTEGER
    ***Note:*** Number of iterations for cross-validation of semi-supervised learning step. This parameter feeds into PyProphet [default: 3]. The default number of cross-validation iterations is 3 in parallel. However, increasing the number of data points (conditions, replicates, and proteome coverage) compared to the sample data may benefit from increasing the number of iterations to increase classifier robustness.
    --ss_initial_fdr FLOAT
    ***Note:*** Initial FDR cutoff for best scoring targets. Feeds into PyProphet [default: 0.1]. A higher initial FDR cutoff will mean that there are more false interactions included. In other words, an FDR of 0.1 will mean that around 10% of the identified interactions are false. Subsequent steps of lowering the FDR will increase the stringency of the classifier with each iteration.
    --ss_iteration_fdr FLOAT
    ***Note:*** Initial FDR cutoff for best scoring interactions of subsequent semi-supervised learning iterations performed by pyProphet [default: 0.05].
    --ss_num_iter INTEGER
    ***Note:*** Number of iterations for semi-supervised learning step performed by PyProphet [default: 10]. For each cross-validation iteration, the default is to perform 10 semi-supervised learning steps to increase the separation of true and false interactions.
    --xgb_autotune / --no-xgb_autotune
    ***Note:*** Autotune hyperparameters after semi-supervised learning by PyProphet [default: no-xgb_autotune]. After semi-supervised learning, this parameter will enable the XGBoost hyperparameters to be tuned using the hyperopt package. Hyperparameters define how the classifier is learned (e.g., number of nodes in a neural network) and are different than the previously generated scores during data preprocessing. This option should only be selected if SECAT is being adapted to data that is very different than the sample (e.g. different co-fractionation methods).
    --parametric / --no-parametric
    ***Note:*** Does parametric estimation of p-values for PyProphet [default: no-parametric]. Instead of an empirical p-value estimation (described later in the pi0_lambda parameter), enabling this parameter will assume a Gaussian distribution of p-values. Typically, this parameter is not necessary unless the data is very different from the sample, and was previously used for the Storey-Tibshirani q-value estimation.^35^
    --pfdr / --no-pfdr
    ***Note:*** Compute positive false discovery rate (pFDR) instead of FDR for PyProphet [default: no-pfdr]. Typically should not be used, but provides all the parameters discussed for the Storey-Tibshirani q-value estimation.^35^
    --pi0_method [smoother|bootstrap]
    ***Note:*** Either "smoother" or "bootstrap" as the method for automatically choosing tuning parameter in the estimation of pi0, the proportion of true null hypotheses. Feeds into PyProphet [default: bootstrap]. Typically, this parameter should not be changed. For more information, refer to the Storey-Tibshirani q-value estimation.^35^
    --pi0_smooth_df INTEGER
    ***Note:*** Number of degrees-of-freedom to use when estimating pi0 with a smoother. Feeds into PyProphet [default: 3]. Typically, this parameter should not be changed. For more information, refer to the Storey-Tibshirani q-value estimation.^35^
    --pi0_smooth_log_pi0 / --no-pi0_smooth_log_pi0
    ***Note:*** If True and pi0_method = "smoother", pi0 will be estimated by applying a smoother to a scatterplot of log(pi0) estimates against the tuning parameter lambda. Feeds into PyProphet [default: no-pi0_smooth_log_pi0]. Typically, this parameter should not be changed. For more information, refer to the Storey-Tibshirani q-value estimation.^35^
    --lfdr_truncate / --no-lfdr_truncate
    ***Note:*** If True, local FDR values greater than 1 are set to 1. Feeds into PyProphet [default: lfdr_truncate]. Typically, this parameter should not be changed. For more information, refer to the Storey-Tibshirani q-value estimation.^35^
    --lfdr_monotone / --no-lfdr_monotone
    ***Note:*** If True, local FDR values are non-decreasing with increasing p-values. Feeds into PyProphet [default: lfdr_monotone]. Typically, this parameter should not be changed. For more information, refer to the Storey-Tibshirani q-value estimation.^35^
    --lfdr_transformation [probit|logit]
    ***Note:*** Either a "probit" or "logit" transformation is applied to the p-values so that a local FDR estimate can be formed that does not involve edge effects of the [0,1] interval in which the p-values lie. Feeds into PyProphet [default: probit]. Typically, this parameter should not be changed. For more information, refer to the Storey-Tibshirani q-value estimation.^35^
    --lfdr_adj FLOAT
    ***Note:*** Numeric value that is applied as a multiple of the smoothing bandwidth used in the density estimation [default: 1.5]. Typically, this parameter should not be changed. For more information, refer to the Storey-Tibshirani q-value estimation.^35^
    --lfdr_eps FLOAT
    ***Note:*** Numeric value that is threshold for the tails of the empirical p-value distribution. Feeds into PyProphet [default: 1e-08]. Feeds into the PyProphet function. Typically, this parameter should not be changed. For more information, refer to the Storey-Tibshirani q-value estimation.^35^
    --plot_reports / --no-plot_reports
    ***Note:*** Plot reports of results, scorer and other PyProphet outputs for all confidence bins [default: no-plot_reports]. This can be useful for generating many figures for manual inspection of the confidence bins creating during data preprocessing.
    --threads INTEGER
    ***Note:*** Number of threads/CPU to use for parallel processing. -1 means all available CPUs [default: −1].
    --export_tables / --no-export_tables
    ***Note:*** Saves two ‘.csv’ tables. One for the target network and its scores used by SECAT’s learning, and another table for the learning networks (positive and negative) with the scores assigned by SECAT. These tables can be used to survey SECAT’s ability to differentiate between targets and decoys and to directly download a full table of the interaction network without going through the SQL file [default: no-export_tables]
    --help
    ***Note:*** Shows brief description of parameters.
    ***Optional:*** Evaluate the learning model. Review the output files from this step to evaluate SECAT’s ability to distinguish between targets and decoys in the data.

7. Proceed to the PPI quantification step for SECAT to assess the differential interactions between two conditions.

***Note:*** If using a single condition, then skip the PPI Quantification step. The plotting and statistics step can be used to explore the interactions recovered in the single condition. Furthermore, if the ‘export_tables’ parameter was selected, then the resulting tables can be used to directly survey the interaction networks and their assigned q-values.

### PPI quantification

**Timing: 30–60 min**

Estimates abundance and interactor ratios of protein interactions and the confidence for differential changes, as described in the original SECAT paper.^1^ This step aggregates peptide-level abundances and reports total abundance, assembled abundance, and monomer abundance at the protein level and interactor abundance, complex abundance, and interactor ratio at the interaction level. Quantitative fold-change values, as well as a z-score values underlying the differential analysis are provided. For more information, please refer to the initial VIPER publication.^19^

8. Run the quantification step using the output SECAT file from the learning step and select the necessary parameters.
  a. Example code from sample data:
    >secat quantify --in=hela_string.secat --control_condition=inter
  b. Required/Important Parameters:
    --in PATH
    ***Note:*** [Required] Input SECAT file from previous step.
    --control_condition TEXT
    ***Note:*** [Required] Specify control condition identifier. Setting this parameter to "center" will compare all conditions against all and use the mean as reference for quantification.
    --maximum_interaction_qvalue FLOAT
    ***Note:*** Maximum q-value for interactions to be considered for quantification [default: 0.05].
    ***Note:*** Make sure that the maximum interaction q-value is not below all the measured q-values. For lower quality data, this metric will need to be increased. Consider the output tables of the q-value distribution to determine whether to increase or decrease the cutoff. Refer to problem 7 in troubleshooting for details. Increasing this value will dramatically increase the coverage at the cost of additional noise. A very large cutoff will result in data that is less mechanistic and closer to a full proteome analysis without fractionation (e.g. as analyzed by mapDIA).^36^
  c. Other parameters
    --out PATH
    ***Note:*** Output SECAT file. Use the out PATH to save this part of the analysis in a new SQLite database. Otherwise, the default is to save the output in the --in PATH.
    --paired / --no-paired
    ***Note:*** Whether replicates should be paired (e.g., replicates 1 of conditions A & B were measured with heavy and light SILAC labels as part of the same runs). Paired replicates need to have the exact same “replicate_id”, defined in the experimental design table [default: no-paired]. This parameter is useful in scenarios where a paired t-test should be conducted instead of an independent t-test.
    --min_abs_log2fx FLOAT
    ***Note:*** Minimum absolute log2 fold-change for integrated nodes. Interaction below the set log2fx will be ignored [default: 1.0]. This value can be increased to focus only on interactions that change dramatically between conditions. It will make differences easier to spot but reduce coverage.
    --minimum_peptides INTEGER
    ***Note:*** Minimum number of peptides required to quantify an interaction [default: 1]. SECAT uses a top N approach and increasing the minimum number of peptides will remove many proteins from analysis. Typically, increasing this parameter does not have many benefits.
    --maximum_peptides INTEGER
    ***Note:*** Maximum number of peptides used to quantify an interaction [default: 3]. Only the highest intensity peptides will be chosen for each protein. Setting a maximum number of peptides will remove less intense peptides that may result in non-confident interactions.
    --missing_peptides TEXT
    ***Note:*** Whether missing peptide abundances should be set to 0 ("zero") or dropped ("drop") for fold change computation [default: zero]. Setting the missing peptide abundances to zero will allow the algorithm to assess changes even in the absence of measured signal. It is recommended to set as zero to include these cases.
    --peptide_log2fx / --no-peptide_log2fx
    ***Note:*** Whether peptide-level log2fx should be computed instead of protein-level. Protein-level is more robust if measured peptides are variable between conditions or replicates [default: peptide_log2fx]. This option was added to allow analysis of some legacy DDA data.
    --threads INTEGER
    ***Note:*** Number of threads/CPU to use for parallel processing. -1 means all available CPUs [default: -1].
    --help
    ***Note:*** Shows brief description of parameters.

9. Proceed to export the results in the next step to view the differential interaction networks.

### Export of results

**Timing: <1 min**

Exports tables of that are formatted for network creation using software such as Cytoscape. To further explore these outputs, refer to Table 3 and the sample data linked in the key resources table.

10. Run the export step using the output SECAT file from the quantification step and select the necessary parameters.
  a. Example code from the sample data:
    >secat export --in=hela_string.secat
  b. Parameters:
    --in PATH
    ***Note:*** [Required] Input SECAT file from previous step.
    --level [bait|interaction]
    ***Note:*** Export either all interactions of bait proteins or individual interactions. This parameter is only considered if --extra is True [default: bait].
    --id TEXT
    ***Note:*** Export a specific Uniprot bait_id (e.g., Q10000) or a specific interaction id (e.g., Q10000_P10000). If no id is provided, all baits or all interactions will be exported depending on the value of --level. This parameter is only considered if --extra is True.
    --max_qvalue FLOAT
    ***Note:*** Maximum q-value to consider interactions for export of the network files. This parameter is used for the default exported data as well as the extra data [default: 0.01].
    --min_abs_log2fx FLOAT
    ***Note:*** Minimum absolute log2 fold-change for integrated nodes. This parameter is only considered if --extra is True [default: 1.0].
    --mode [quantitative|detection]
    ***Note:*** Select mode to order interaction export tables by. This parameter is only considered if --extra is True [default: quantitative]
    --combined / --no-combined
    ***Note:*** Select interactions and baits according to combined q-values. This parameter is only considered if --extra is True [default: no-combined].
    --peptide_rank INTEGER
    ***Note:*** Select the number of most intense peptides to export. This parameter is only considered if --extra is True [default: 6].
    --extra BOOLEAN
    ***Note:*** Choose whether or not to export raw ‘.csv’ files of data used in ‘secat plot’ [default: False].
    --help
    ***Note:*** Shows brief description of parameters.

11. Pot the network of this step using Cytoscape or other network building software. Refer to the key resources table for a walkthrough on how to build these networks using Cytoscape.

***Note:*** The export step does not edit the SECAT file, and all following steps can be performed regardless of whether this step was performed.

**Table 3:** Output files

File name (E.g., experiment X)	Description
Experiment_X.secat	SQLite format file containing all saved databases created and edited throughout the SECAT run.
Experiment_X_raw.pdf	A plot of total peptide intensities summed by fraction before SECAT lowess normalization, produced during the preprocessing step.
Experiment_X_norm.pdf	Plot of total peptide intensities summed by fraction after SECAT lowess normalization, produced during the preprocessing step.
Experiment_X_count.pdf	Plot of peptide counts by fraction, produced during the preprocessing step.
Experiment_X_learning.pdf	General outputs related to the pyProphet semi-supervised learning, produced during the learning step.
Experiment_X_learning_score.pdf	More detailed outputs related to the pyProphet semi-supervised learning, produced during the learning step.
Experiment_X_net_int_scored.csv	Table of all the protein interactions in the target network and their scores/metrics, produced during the learning step.
Experiment_X_learn_int_scored.csv	Table of the protein interactions used in the learning networks and their scores/metrics, produced during the learning step.
Experiment_X_differential_network.csv	Differential network table of the protein interactions and related values, produced during the export step.
Experiment_X_differential_nodes.csv	Differential protein table of network nodes and related values, produced during the export step.
Experiment_X_differential_nodes_level.csv	Differential protein table of network nodes and related values separated by differential level, including proteoVIPER scores, produced during the export step.
Experiment_X_differential_edges.csv	Differential interactions of network edges and related values, produced during the export step.
Experiment_X_differential_edges_level.csv	Differential interactions table of network edges and related values separated by differential level, including proteoVIPER scores, produced during the export step.
Experiment_X_differential_proteins_level.csv	Differential protein table of all protein nodes produced during the export step.
Experiment_X.txt	Text file with general overview of analysis statistics, including peptide, protein, interaction, and differential numbers, produced during the statistics step.
Experiment_X_AAAAAA_BBBBBB.pdf	PDF file plotting two proteins and their interaction scores, produced during the plotting step.
Experiment_X_AAAAAA_BBBBBB_peptide.csv	Table of raw peptide intensities and metadata, produced during the export step if –extra=True and the –level=interaction.
Experiment_X_AAAAAA_BBBBBB_feature.csv	Table of supplementary data, produced during the export step if –extra=True and the –level=interaction.
Experiment_X_AAAAAA _peptide.csv	Table of raw peptide intensities and metadata, produced during the export step if --extra=True and the --level=bait.
Experiment_X_AAAAAA _feature.csv	Table of supplementary data, produced during the export step if --extra=True and the --level=bait.

### Plotting of chromatograms

**Timing: <1 h**

Prints PDF files plotting the protein elution profiles. Proteins and interactions of interest can be selected by uniprot ID or significance.

12. Run the plotting step using the output SECAT file from the learning or quantification step and select the necessary parameters.
  a. Example code from the sample data:
    >secat plot --in=hela_string.secat
  b. Required/Important parameters:
    --in PATH
    ***Note:*** [Required] Input SECAT file from either the learning or quantification step.
    --level [bait|interaction]
    ***Note:*** Plot either all interactions of the selected bait proteins or individual interactions between the proteins that fit the criteria [default: bait].
    --id TEXT
    ***Note:*** Plot specific UniProt bait_id (Q10000) or interaction_id (Q10000_P10000).
    --max_qvalue FLOAT
    ***Note:*** Maximum q-value to plot baits or interactions [default: 0.01].
    ***Note:*** Adjust based on data, often a reason for not getting plots is that the max_qvalue needs to be increased for lower quality data. To know the relative q-value range of interest, one can assess the q-value range of the reported interactions.
    --peptide_rank INTEGER
    ***Note:*** Number of most intense peptides to plot [default: 6].
  c. Other Parameters:
    --min_abs_log2fx FLOAT
    ***Note:*** Minimum absolute log2 fold-change for integrated nodes [default: 1.0].
    --mode [quantitative|detection]
    ***Note:*** Select mode to order interaction plots by [default: quantitative]. Quantitative: will select scores from the “secat quantify” module of SECAT analysis. Detection: will select interactions to plot based on the “secat learn” module of the SECAT analysis (e.g., useful if only a single replicate/condition is measured).
    --combined / --no-combined
    ***Note:*** Select interactions and baits according to combined q-values [default: no-combined]. The combined q-value will be more robust, but if there are big differences between conditions then certain interactions may be missed.
    --help
    ***Note:*** Shows brief description of parameters.

13. View the exported plots of the selected protein interactions.

***Note:*** The plot step does not edit the SECAT file, and all following steps can be performed regardless of whether this step was performed.

### Report of statistics

**Timing: <1 min**

Saves a text file of the general statistics from the various stages of SECAT analysis. For example, the number of proteins, peptides, and interactions at a certain FDR.

14. Run the statistics step using the output SECAT file from any of the SECAT steps. Information is added to the exported text file in the preprocessing, learning, and quantification steps.
  a. Example code from the sample data:
    >secat statistics --in=hela_string.secat
  b. Parameters:
    --in PATH
    ***Note:*** [Required] Input SECAT file following any step past preprocessing.
    --min_abs_log2fx FLOAT
    ***Note:*** Minimum absolute log2 fold-change for integrated nodes [default: 1.0].
    --help
    ***Note:*** Shows brief description of parameters.

15. View the text file to find the number of peptides, protein, and interactions for each condition/replicate.

## Expected outcomes

**Figure 3:**
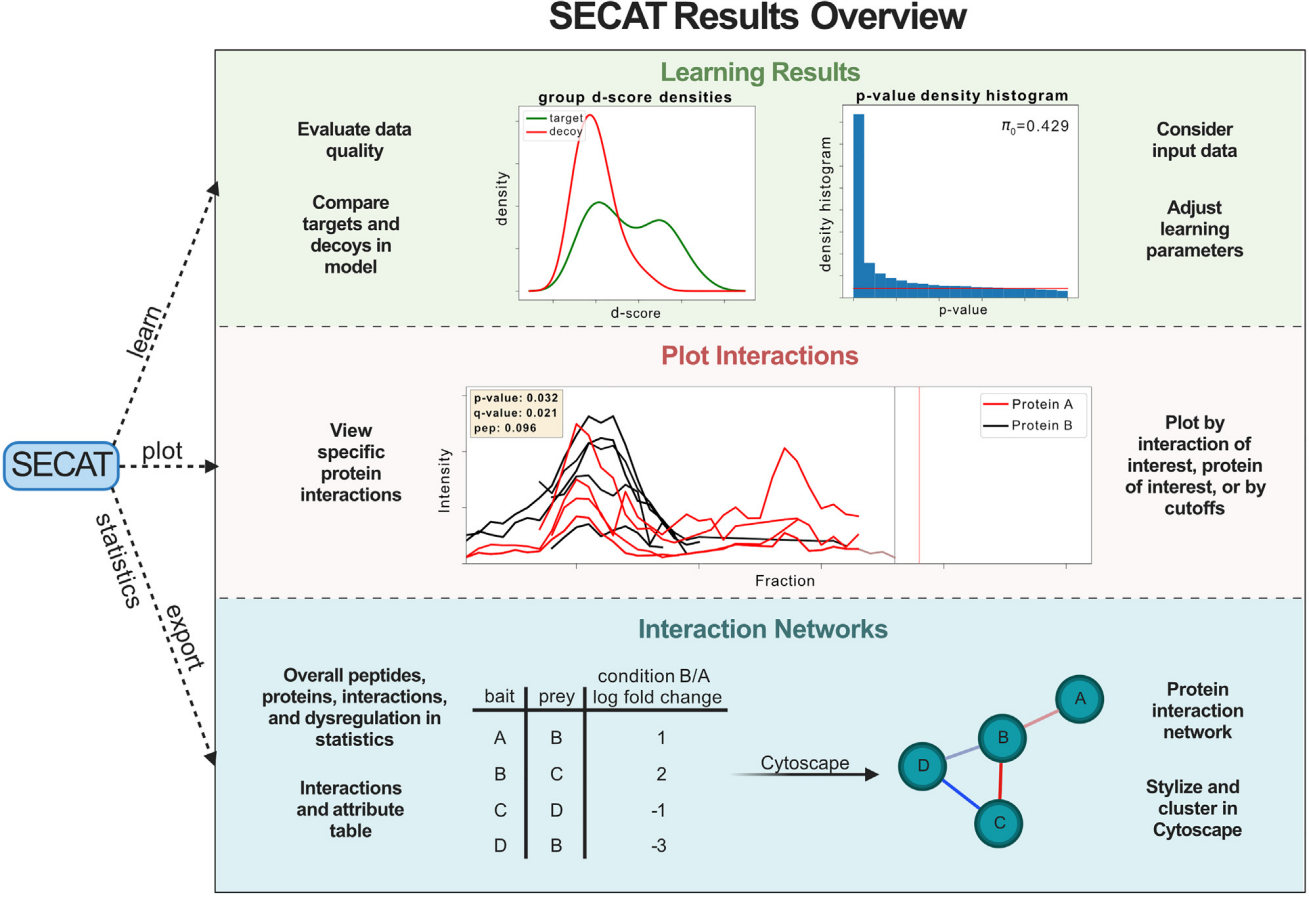
SECAT results overview SECAT has outputs relating to semi-supervised learning, such as d-score (discriminant score) distribution and p-value density that can be used to gauge the quality of the data and classifier. Using the plot function, SECAT allows plotting of peptide-level individual interactions of interest, with several parameters for selecting specific and numerous interactions for plotting. To get an overview of the data, a statistics file can be output as a text file for viewing overall numbers of peptides, proteins, interactions, and dysregulation at multiple q-values. Lastly, multiple ‘.csv’ files are exported with the ‘export’ function that can be used to create interaction networks.

The SECAT analysis is split into multiple functions that perform different operations and evaluations on the co-fractionation data. As shown in Figure 3, the analysis can be evaluated at different stages of the analysis. The data quality can be evaluated by viewing the normalization plots (data preprocessing) and learning statistics (learning). SECAT provides easy access to the overall numbers of peptides, proteins, and interactions with the statistics function. Protein interactions can be viewed individually with the plot function, and differential protein interactions networks can be built using the exported tables and Cytoscape. Overall, SECAT provides a suite of tools to analyze the quality of co-fractionation data to building differential interaction networks (Table 3).

## Limitations

The analysis options and insights will be limited for lower quality data. Identification of significant interactions will be limited by low SEC resolution, low proteome coverage, and the power of the experimental design. Furthermore, the analysis relies heavily on the pre-knowledge databases provided and has limited capability for the inference of novel interactions. Additionally, the differential analysis will be limited to two conditions at a time and will depend on the strength of the biological signal or perturbation. Given that SECAT analyzes pairwise interactions only, one will need to refer to outside the software package to infer protein complexes.

## Troubleshooting

### Problem 1

General issues with SEC experimental setup.

Here are some common issues that many experience while setting up a SEC-MS experiment.

• Lack of protein complex separation, specifically in the higher molecular weight fractions.
• Loss of protein complex integrity during co-fraction.
• Misalignment or loss of fractions between conditions and/or replicates. For example, separate co-fractionation experiments with different standards may result in the same complex eluting in different fractions or change the shape/size of a complex’s elution profile.

If necessary, be sure to address these before you begin.

### Potential solution

For most issues regarding setting up a SEC-MS experiment, we recommend reviewing the Bludau et al. (2020) experimental design section.^12^ In regard to considerations for setting up a quantitative SEC experiment with multiple conditions and replicates, refer to Oberg & Vitek.^37^

Some additional considerations to consider are the molecular weight range of the co-fraction apparatus used. For example, if certain complexes of interest are above the upper limit of the apparatus’ range, then the complex may co-elute with many other complexes in the so called, void region. The void region may contain many complexes and detract from the complex’s resolution and result in many false positive interactions.

Furthermore, misalignment and loss of fractions is a common result of large-scale co-fractionation-MS experiments. In our experience, conditions and replicates should be run in a completely random manner for small experiments to minimize biases introduced in different conditions and replicates. Additionally, co-fractionation runs should be run with intermittent MW standards and make use of the same fractions as conducted with the sample data. However, if an unexpected problem does occur, then SECAT will automatically realign the fractions if each fraction is annotated with the appropriate approximate molecular weight in the cofractionation molecular weight annotation file. Additionally, interaction identifications are performed in SECAT on a condition/replicate basis. Therefore, changes in peak location and shape between conditions/replicates will not affect the intra-run interactions identifications.

### Problem 2

Selecting a single UniProtKB identifier.

Many MS analysis software solutions provide protein groups or lists of proteins and protein isoforms in association with a single peptide. However, SECAT requires a single UniProtKB identifier for each peptide identification and does not consider multiple protein isoforms. If necessary, be sure to address this problem when formatting the input data.

### Potential solution

Ideally, users can make use of a single ‘most-confident’ or ‘razor’ protein identification. In which case, users should ignore the protein group or list of identifications and select the most highly confident protein ID. However, if this is not possible, users are recommended to select either the canonical UniProtKB ID or the ID of interest during more targeted analyses. A simple method to circumvent this issue is to run the analysis software using a UniProt FASTA database that only contains canonical protein IDs.

### Problem 3

Intensity files.

During data preprocessing, users may encounter an error relating to SECAT’s inability to find specific columns or tables. For example:

ValueError: could not convert string to float: 'XXXXXXX'

preprocess.py", line 347, in read_matrix

df['peptide_intensity'] = df['peptide_intensity'].apply(float)

Execution failed on sql 'SELECT protein_id, protein_mw FROM PROTEIN;': no such table: PROTEIN

or

KeyError: 0

no such table: PROTEIN

File "∼/.local/lib/python3.7/site-packages/secat/preprocess.py", line 352, in read_matrix

if "/" in df['protein_id'][0]:

### Potential solution

This is likely a problem will the column names of the input files. Check the column names of your intensity and annotation files and make sure they correspond. A common fix is to make sure that the run_id in the --sec file matches the column names in the intensity files. Additionally, check that the columns of the tables are in the same order as the sample data or that the preprocess --column setting is used correctly.

### Problem 4

Uniprot parsing.

During data preprocessing, users may encounter an error when importing their own uniprot XML files. This is to be expected if the user is analyzing a less common model organism. An error such as this may show up:

Info: Parsing UniProt XML file uniprot_XXXX_YYYYYYYYY.xml.gz.

Info: Parsing network file XXXX.protein.links.v11.0.txt.gz.

File "∼/.local/lib/python3.7/site-packages/pandas/core/reshape/merge.py", line 1252, in _maybe_coerce_merge_keys raise ValueError(msg)

ValueError: You are trying to merge on object and float64 columns. If you wish to proceed you should use pd.concat

### Potential solution

This is a problem with SECAT not being able to parse the used UniProtKB XML file. As previously discussed, SECAT has been tested for parsing Human, Mouse, and Yeast files, but other organisms may not be compatible with the current version of SECAT. Resolving this issue would first require making sure that the latest version of the UniProtKB XML file is being used, and beyond that one must modify the ‘preprocess.py’ module in the Python package. Uniprot files with different IDs and structures may require editing in the SECAT’s parser to extract the correct fields.

### Problem 5

Normalization problem.

During data preprocessing, SECAT’s normalization may have issues with data that has very low intensity or missing fraction data. For reference, compare the SECAT preprocess output of the normalized vs. raw pdf files. A normalization problem may look something like this:

**Figure:**
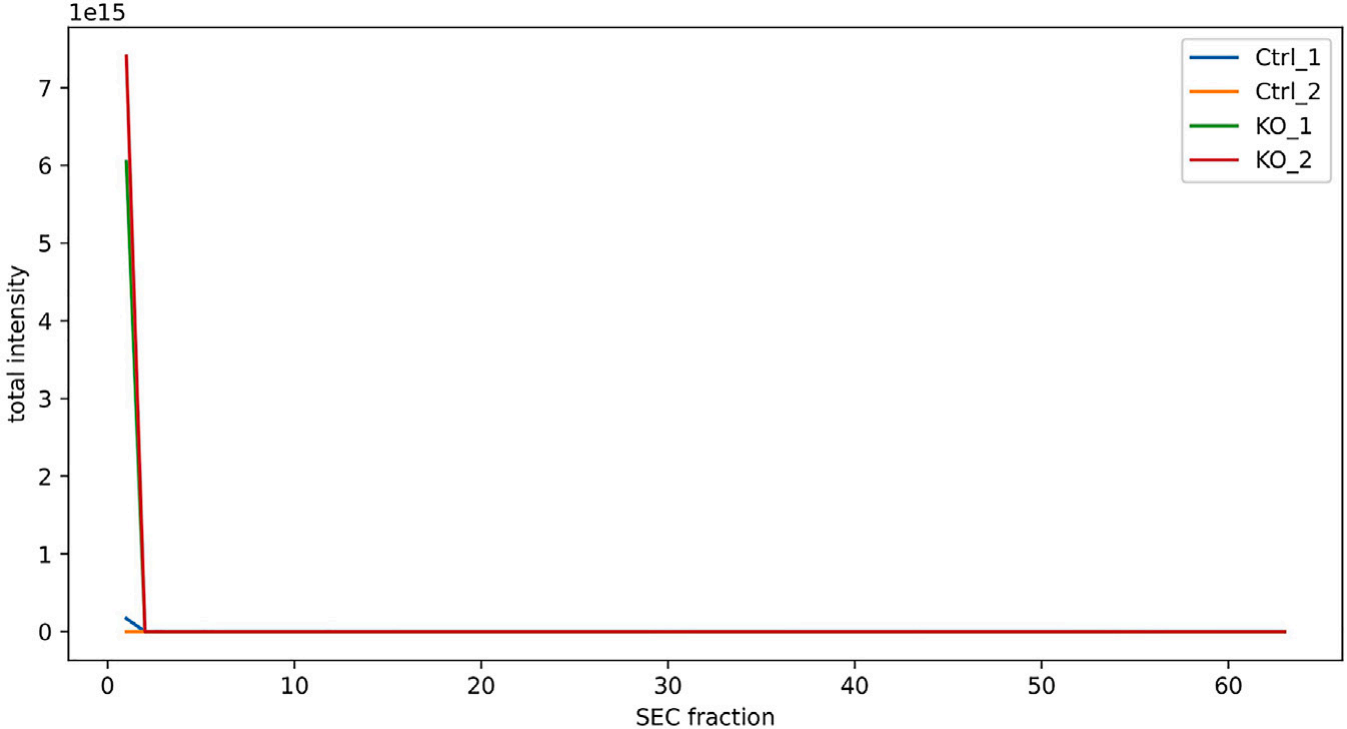

### Potential solution

One option is to perform SECAT analysis without normalization. In ‘secat preprocess’ use parameter ‘--no-normalize’. Refer to the data preprocessing step for more information.

A second solution is to remove specific fractions from the SECAT analysis. This is a promising solution if there are empty fractions as shown below. Note that the empty fractions only need to be removed from problematic conditions/replicates because SECAT will realign fractions between all conditions/replicates based on the co-fractionation annotation file.

**Figure:**
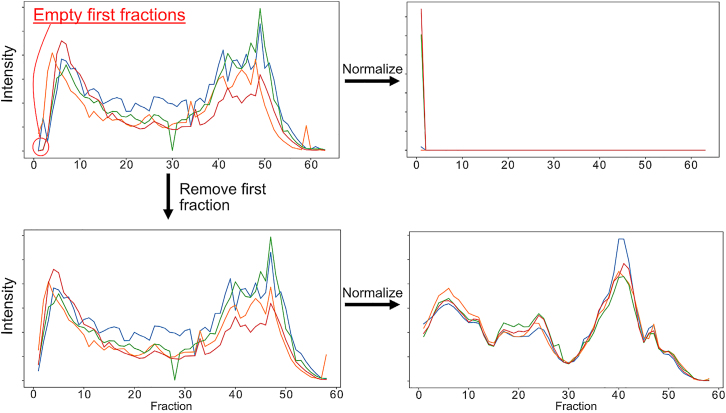

### Problem 6

Estimation of pi0 through lambda tuning function fails.

During the PPI detection step, SECAT sometimes gives errors relating to estimating a pi0 value and is incapable of detecting interactions. For example:

Error: The estimated pi0 <= 0. Check that you have valid p-values or use a different range of lambda.

sqlite3.OperationalError: no such table: FEATURE_SCORED

pandas.io.sql.DatabaseError: Execution failed on sql 'SELECT ∗, condition_id || "_" || replicate_id AS tag, bait_id || "_" || prey_id AS interaction_id FROM FEATURE_SCORED;': no such table: FEATURE_SCORED

### Potential solution

If semi-supervised learning in PyProphet fails at this step, failed tuning of the lambda function is typically not the issue but rather the symptom. Most often, the quantitative consistency or coverage of the dataset is not sufficient for SECAT. In this case, manual inspection and data quality assessment as described is recommended.^12^ For debugging purposes, but not production-level applications, e.g., to visualize the data using SECAT, the lambda function could also be set to an extremely low value (e.g., 0.01 or 1e-6), which result in a pi0 estimate close to 1.0, thus assuming that almost all candidate PPIs result in false signals.

In some cases, the semi-supervised learning in PyProphet fails due to low quality and consistency of the data. To rule this out, users should first check the protein identification coverage. In humans, each replicate of a condition should contain at least 3,000 or preferably more than 5,000 identified proteins. Given sufficient protein identification coverage, users should also check the consistency of number of recovered peptides per fraction, as well as the normalized and raw total intensities plots that are output from the data preprocessing step. Each replicate should have similar elution shapes and fractions should be non-staggered.

If data quality and consistency issues can be ruled out, by the rules above or by peptide sibling correlation, as described previously,^12^ it might be useful to tweak these parameters, considering the following assumptions:

To compute confidence scores for each interaction, PyProphet needs to estimate the proportion of truly absent interactions (pi0) that still result in a partially overlapping signal using the lambda tuning function, proposed by Storey & Tibshirani (2003).^35^ The default start, end and step values for the lambda function are defined as three parameters separated by an empty space. [default: 0.01, 0.5, 0.01] Alternatively, a fixed value can be set as described below.

Either use:

• <START END STEPS>, e.g., 0.1, 1.0, 0.1 (set as: 0.1 1.0 0.1).
• set to fixed value, e.g., 0.4 (set as: 0.4 0 0).

For high proteome coverage, high quantitative consistency datasets as the provided examples, this parameter typically does not need to be modified. For some scenarios, it might be useful to enforce a more conservative estimate of pi0 by setting a fixed value (e.g., 0.4) or by limiting the range of lambda (e.g., from 0.01–0.1). A detailed description of the assumptions and considerations can be found in Fig. 3 of Storey & Tibshirani (2003).^35^

### Problem 7

Quantify interactions.

During PPI quantification, SECAT may provide value errors when creating data frames. For example:

File "∼/.local/lib/python3.7/site-packages/secat/quantify.py", line 140, in quantify_complexes

complexes_sec = complexes.groupby(['condition_id','replicate_id','bait_id','prey_id']).apply(sec_summarize).reset_index(level=['condition_id','replicate_id','bait_id','prey_id'])

ValueError: cannot insert prey_id, already exists

### Potential solution

SECAT may get an error from not having any interactions make the q-value cutoff. One solution is to increase the maximum_interaction_qvalue but increasing the q-value cutoff will increase the likelihood of recovering false interactions.

## Resource availability

### Lead contact

Further information and requests for resources and reagents should be directed to and will be fulfilled by the lead contact, George Rosenberger (gr2578@cumc.columbia.edu).

### Materials availability

This study did not generate new unique reagents.

### Data and code availability

The sample datasets used in this protocol are available at Zenodo Data: https://doi.org/10.5281/zenodo.3515927. The SECAT code used for this protocol are available at Zenodo Data: https://doi.org/10.5281/zenodo.7799334.

Cytoscape walkthrough for network analysis is available at Mendeley Data: https://doi.org/10.17632/xkrrw8xtnj.1.
